# Elementary students’ social, emotional, and cognitive development during the COVID- 19 pandemic in North America: A scoping review

**DOI:** 10.1371/journal.pgph.0005148

**Published:** 2025-09-11

**Authors:** Julia Yates, Cole Young, Tara Mantler

**Affiliations:** 1 Health and Rehabilitation Sciences, The University of Western Ontario, London, Ontario, Canada; 2 School of Health Studies, The University of Western Ontario, London, Ontario, Canada; University of Tunis El Manar Faculty of Medicine of Tunis: Universite de Tunis El Manar Faculte de Medecine de Tunis, TUNISIA

## Abstract

The COVID- 19 pandemic and subsequent disruptions to in-person learning presented unanticipated challenges to elementary school systems across North America. Elementary students are particularly vulnerable to the negative outcomes of extended online learning, as this developmental stage is crucial for the social, emotional, and cognitive growth of students. To date, no research has synthesized what we currently know about the impacts of the pandemic on these developmental outcomes among students. As such, this scoping review explores and synthesizes what is currently known regarding elementary students’ social, emotional, and cognitive development during the COVID- 19 pandemic in North America. In accordance with the Joanna Briggs Guide, three databases were searched in September 2024, and results were uploaded into Covidence wherein they were screened for eligibility. A total of 13 peer-reviewed articles met the inclusion criteria for this review (i.e., included elementary students, spoke to social/emotional/cognitive development, peer-reviewed articles written in English and conducted in North America). Thematic analyses revealed four themes including (1) lack of socialization opportunities; (2) emotional regression; (3) cognitive gaps in learning; and (4) inequitable impacts on development. School-based supports are needed for all students, but particularly those facing multiple, intersecting health and social disparities, to bolster positive development post-pandemic and to mitigate long-term negative health outcomes.

## Introduction

The COVID- 19 pandemic presented unanticipated challenges to elementary school systems across North America, with significant disruptions to in-person learning that impacted the development of elementary students. The implementation of social distancing strategies, which have been found to impact children’s social, emotional, and cognitive development [1], affected approximately 5.1 million elementary students in Canada, 33.5 million in Mexico, and 61.7 million in the United States by 2021 [2]. Throughout the duration of the pandemic, elementary students faced a near-constant oscillation between emergency remote learning and in-person learning. The duration of emergency remote learning for elementary students (students aged 5–12 years of age) varied by country due to differing pandemic mandates, with students experiencing an average of 17, 26, and 25 weeks of emergency remote learning in Canada, Mexico, and the United States, respectively, within the first year of the pandemic [3–5]. Beyond the first year of the pandemic, in-person school closures persisted with North American schools reporting an average of 535 days of combined full and partial closures [6]. The duration of the closures varied across regions, with some areas prioritizing in-person schooling for elementary learners and others prioritizing public health [3]. Resultingly, some elementary children experienced declines and/or deficits in their developmental trajectories, including their social, emotional, and cognitive skills – all of which experience significant changes during the key developmental time period of elementary school. The developmental impacts of these prolonged closures over a three-year span have begun to be described within literature, with the evidence overwhelmingly pointing to widespread cognitive development losses resulting with particularly sharp declines in student achievement in core subjects such as literacy and numeracy [7,8]. Together, this highlights that the pandemic was one of the most severe educational crises in recent decades [9].

It is established that school and in-person interactions are fundamental to elementary student development, with classroom engagement serving as a key mediator in helping students establish positive attitudes about education [10,11]. The transition to online learning during the pandemic disrupted classroom routines, altering interactions between teachers and students, which are understood to be vital for student development [12]. Teachers play a pivotal role in adapting instructions and educational materials to align with the individual students’ abilities and interests, thereby creating dynamic and responsive learning environments [12]. This further underscores a concern within the context of the pandemic, as a growing body of literature highlights the academic challenges faced by elementary educators due to pedagogical limitations, including difficulties in motivating students, maintaining engagement, and overcoming technological barriers associated with online instruction [8].

It is not surprising that lower-grade elementary students, ages 5- to 8-years-old, are particularly vulnerable to the negative outcomes of extended online learning. This developmental stage is crucial for the social, emotional, and cognitive growth of students, in which childhood education, including play and inquiry-based instruction, plays a key role in developing skills through socialization that are necessary for students’ future academic success [7,12]. The decline in peer interactions and group activities during remote learning significantly restricted students’ collaborative learning experiences, with anticipated long-term effects on socioemotional, cognitive, and linguistic development [10,7]. While all elementary students experienced the impacts of the COVID- 19 pandemic, these impacts may not have been felt uniformly amongst students. Namely, students from marginalized groups who faced pre-existing developmental vulnerabilities, such as those students from racialized or low socioeconomic status families, may have experienced heightened impacts of the pandemic given their multiple, intersecting developmental inequities [13]. Research has also established that online schooling does not effectively substitute in-person interactions, which are essential for all students to build identity, social knowledge, competence, and practice social skills. These skills are fundamental to social development, and inadequate learning can contribute to aggression, peer conflict, and reduced social competence; with negative psychological and physical health implications [14]. Therefore, understanding the pandemic’s lesser-known impact on social learning in North America is crucial for addressing potential development deficits that emerged during periods of online learning.

Recent literature has begun to explore the pandemic’s effects on developmental outcomes. A systematic review by Jones and colleagues [15] of early childhood (children aged 0–8 years) during the pandemic reported higher rates of developmental delays, social and emotional challenges, language challenges, and difficulty mastering learning milestones compared to pre-pandemic. However, a synthesis of the effects of the pandemic on elementary students, aged 5–12 years, remains unexplored [16].

Recent literature has given considerable attention to understanding teachers’ difficulties during the pandemic; however, there remains a lack of research on how the impact of these challenges, particularly the disruptions to student interactions, have affected student developmental outcomes. While existing research in North America has explored the pandemic’s effects on aspects of child development individually, including social, emotional, and cognitive growth, there remains limited literature that comprehensively compares the pandemic’s broad impact on each developmental outcome among elementary students during this critical stage of growth. As such, this scoping review explores the question: “What is currently known regarding elementary students’ social, emotional, and cognitive development during the COVID- 19 pandemic in North America?”. By synthesizing this information, this review hopes to provide insights for key stakeholders in education to help understand the potential long-term effects of the pandemic on these developmental outcomes and better support elementary students following the pandemic. Given that we were interested in identifying what is known in this research area, a scoping review was a more suitable choice than a systematic review which tends to address feasibility, appropriateness, meaningfulness, or effectiveness of a treatment or practice [17].

## Methods

This scoping review adapted the Joanna Briggs Institute 2020 Guide to conducting scoping reviews [18], chosen for its methodological guidance to uphold transparency and rigor in the review process [19]. The Joanna Briggs Institute 2020 guideline consists of six phases: (1) formulating the research question, (2) identifying pertinent studies, (3) selecting studies, (4) organizing the data, (5) compiling, summarizing, and presenting the findings, and (6) consultation. This guideline extends beyond the work of Arksey and O’Malley [20] and Levac and colleagues [21]. Covidence systematic review software (2024) [22] was used to manage the review process, including abstract screening, full-text review, and data extraction. Covidence is a web-based platform that facilitates collaboration among multiple reviewers by streamlining tasks such as screening, data extraction, and resolving disagreements. This review’s findings are presented per the Preferred Reporting Items for Systematic Reviews and Meta-Analyses (PRISMA-ScR) guidelines, adapted for scoping reviews [23]; S1 Checklist).

### Search strategy and information sources

The search strategy was determined in consultation with a librarian from the host institution. Search terms included those related to three overarching concepts (1) elementary students, (2) social, emotional, and cognitive development, and (3) the COVID- 19 pandemic (see S1 Table). The search strategy was run across three databases (i.e., MEDLINE, SCOPUS, and PsycINFO) during September 2024, yielding a total of 575 citations.

### Eligibility criteria

In accordance with the Joanna Briggs Guide, to be included in this review, papers needed to align with the pre-defined elements of the inclusion/exclusion criteria for this review, according to the Participants/Concept/Context framework (PCC). *Participants* were eligible if studies included elementary students in grades kindergarten to 6 (i.e., children between the ages of 5–12 years), or educators/parents speaking on behalf of or regarding elementary students. The *concept* of interest included results pertaining to the social, emotional, and/or cognitive development of elementary students in North America. In this review, working definitions of social, emotional, and cognitive development were established to guide the research process: *social development* considers the processes through which children learn to interact with others around them and how a child develops friendships and other relationships, as well as how they handle conflict with peers (SCAN of Northern [24]); *emotional development* refers to the emergence of the experience, expression, understanding, and regulation of emotions [25]; and *cognitive development* refers to a child’s ability to perform simple to complex mental tasks, including attention, mental processing speed or alertness, thinking skill, problem solving and judgement [26]. Finally, the *context* criteria involved peer-reviewed articles written in English of various designs where the research was conducted within North America during the COVID- 19 pandemic. For the purposes of this review, the COVID- 19 pandemic included the timeframe between March 2020 (i.e., when the World Health Organization declared a public health emergency of international concern; World Health Organization, 2020) [27] and May 2023 (i.e., when the World Health Organization shifted COVID- 19 to an ongoing health issue instead of public health emergency; World Health Organization, 2023) [28]. The North American context was focused on given the differences in COVID- 19 responses around the world which could substantially alter developmental outcomes among children. Articles were excluded if they did not align with any component of this PCC framework.

### Selection of sources of evidence

**Fig 1:**
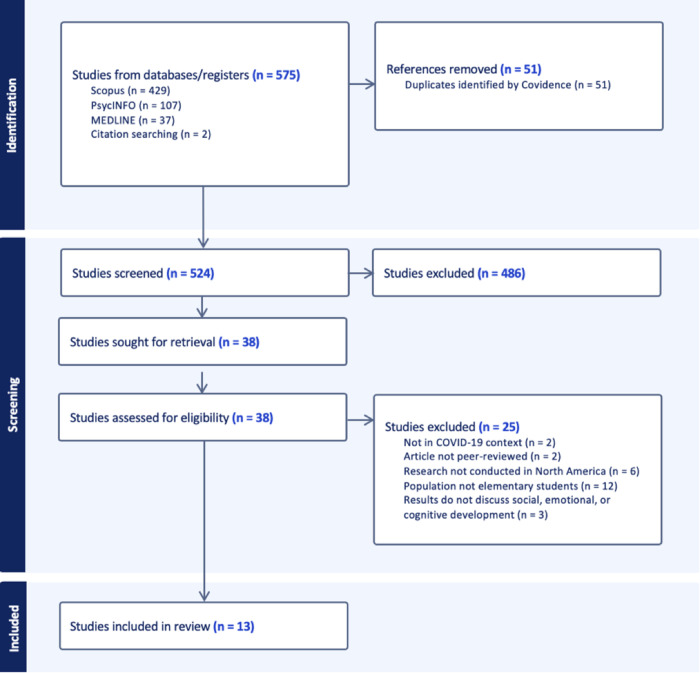
Adapted PRISMA Flow Chart by the Authors.

Two team members (JY and CY) independently completed title/abstract screening (Inter-rater reliability = 0.89) and full-text screening (Inter-rater reliability = 0.95), indicating substantial reviewer agreement across all stages (see Fig 1 for PRISMA diagram). Any disagreements were discussed among team members until consensus was reached.

### Data extraction

For each study, one reviewer completed data extraction using the data extraction template designed by the research team. Information included article author(s), publication year, location of study, sample size, demographic characteristics (i.e., age, sex/gender), study design, assessment of social, emotional, or cognitive development, and important findings.

### Data analysis

Descriptive statistics were used to summarize demographic variables (location, sample size, age, sex/gender). A qualitative inductive thematic analysis was employed to explore and interpret data [20,21,29] while also examining the commonalities and differences in the social, emotional, and/or cognitive development of elementary students in North America during the COVID- 19 pandemic. To complete thematic analysis, the research team first immersed themselves in the data. Then, coding of the data began by identifying relevant segments of the data. Codes were then grouped into broader themes to capture meaningful patterns in the data. Themes were then refined as necessary, and consensus was reached amongst the research team, which included experts in child development, on theme names that could accurately capture the essence of each.

## Results

Preliminary database searches yielded 575 citations. After duplicates were removed, 524 studies were included in title/abstract screening, with 486 studies being excluded at this stage. The full-text of the remaining 38 studies were screened for eligibility, resulting in the exclusion of 25 studies. Thirteen articles met the inclusion criteria and were included in this review.

### Study characteristics

A summary of the 13 included studies is presented in Table 1. The studies included in this review represented 956,436 children (M_age_ = 9.1 years; i.e., fourth grade), 7,449 parents, and 139 elementary educators. Most studies included students of all elementary grades, however two studies focused specifically on kindergarten educators [7,36]. Of the studies that reported the gender of children, most were evenly distributed between females and males. No studies included information on gender diverse children. Five studies were conducted in Canada [7,12,14,33,37], eight studies were conducted in the United States [8,16,36,30–32,34,35], and none were conducted in Mexico. Ten studies discussed social development, five discussed emotional development, and four and cognitive development, respectively. Most studies were conducted during 2020, the first year of the pandemic.

**Table 1:** Summary Characteristics of Included Studies.

Authors, Year	Location	Study Design	Study Timing	Type of Development Explored	Sample Description		
					Sample Size	Mean age of children in years (*SD*)	% Female children
[30]	USA (Boston and Philadelphia)	Cross-sectional mixed methods.	December 2020-May 2021	Emotional	131 children	9.1 (1.16)	48.85%
[16]	USA (Yale University area)	Cross-sectional quantitative.	January-September 2019 and March-June 2020	Social	112 youths	11.8 (2.13)	55.36%
[31]	USA (All 50 States)	Cohort study.	2019-2020 and 2020–2021 school years	Cognitive	955,816 children in grades 1–6	N/A	48.70%
[14]	Canada (Quebec)	Cross-sectional qualitative.	May-June 2020	Social	56 children	8.9 (2.64)	54%
[32]	USA (Michigan)	Cross-sectional quantitative.	February-March 2021	Social and Emotional	305 parents and elementary school-aged children	7.1 (1.6)	N/A
[33]	Canada (Alberta and Manitoba)	Cross-sectional qualitative.	December 2020-February 2021	Social	16 children 15 parents	11.2	N/A
[34]	USA (King County, Washington)	Cross sectional, quantitative.	2021	Social and Cognitive	7,033 parents	5.8	49.3%
[7]	Canada (Ontario)	Cross-sectional mixed methods.	Retrospective reports on pandemic.	Social and Cognitive	92 kindergarten educators	N/A	N/A
[35]	USA (Baltimore City area)	Cohort study.	April-August 2020	Emotional and Cognitive	45 parents	7.5 (2.6)	51.1%
[12]	Canada (Ontario)	Cross-sectional qualitative.	During pandemic.	Social and Emotional	25 elementary teachers 11 parents	N/A	N/A
[8]	USA	Cross-sectional qualitative.	2020-2021 school year	Social	19 elementary teachers	N/A	84%
[36]	USA	Cross-sectional qualitative.	Fall 2020	Social and Emotional	3 kindergarten teachers 4 parents	N/A	N/A
[37]	Canada	Cross-sectional mixed methods.	Spring 2020	Social	36 parents	11.0	N/A

Study timings reflect information provided within each article. Specific dates are provided where possible.

### Thematic findings

Four themes related to students’ development during the COVID- 19 pandemic were revealed, including (1) lack of socialization opportunities- changes to friendships, shifting attitudes towards schooling, decrease in socioemotional skill development; (2) emotional regression resulting from pandemic realities; (3) cognitive gaps in learning; and (4) inequitable impacts on development. See Table 2 for a summary of important findings from each article.

**Table 2:** Data Extraction Chart.

Authors, Year	Purpose	Assessment of Social, Emotional, or Cognitive Development	Important Findings Related to Social, Emotional, or Cognitive Development
[30]	To better understand how social input and context impact emotion recognition, this study investigated emotion recognition in children using images of both masked and unmasked emotional faces.	Emotional development assessed via children completing the DART emotional recognition task which includes static images of actors (child or adult) displaying one of five emotions: happy, sad, angry, fearful, or neutral.	- Significant relationship between age of child and emotion recognition accuracy for unmasked (*r = *0.33, *p* < 0.001) and masked faces (*r =* 0.19, *p* < 0.05) - Masking was related to lower emotion recognition accuracy (*b* = −0.58, *z* = −6.57, *p* < 0.001) - Emotion recognition was worse for sad compared to happy, fearful, angry, and neutral faces (χ^2^(1) = 37.34, *p* < 0.001) - The effect of masking was related to lower emotion recognition accuracy in families reporting more social disruption (*b* = −0.34, *p* = 0.04)
[16]	To investigate whether the associations between different social contexts changed during COVID- 19.	Social development assessed via changes in person-level associations between social interactions during COVID- 19.	- For younger participants, there was a significant decrease in number of positive interactions with friends from prior to during COVID- 19 (*t*(51) = 4.86, *p *< 0.001) - Positive interactions with mothers were the most frequent, followed by fathers (*t*(51) = 3.24, *p *= 0.002) and siblings (*t*(51) = 5.77, *p *< 0.001) - Least frequent positive interactions were with friends (*t*(51) = 1.48, *p *= 0.146) - Negative interactions with siblings were the most frequent, followed by negative interactions with mother (*t*(51) = 4.35, *p* < 0.001) - The associations between negative interactions among social networks increased during COVID- 19
[31]	To compare the reading skills of students in first through sixth grades from the fall of the 2020–2021 school year with the previous 2019–2020 school year.	Cognitive development assessed via the Reading Composite Score (RCS) which provides an estimate of students’ early literacy skills or reading proficiency and is highly related to future reading outcomes. Students were also placed in benchmark categories reflecting (a) their likelihood of reaching later reading outcomes and (b) the recommended support level for students to meet future reading outcomes and goals.	- Impact of pandemic on RCS were significantly negative across all grades, indicating lower overall reading performance during fall 2020–2021 (*p* < 0.001) - Effects were largest for earlier grades (i.e., more noticeable in grade 1 and least in grade 6), suggesting the effect of the pandemic on reading skills was experienced most acutely by younger students, who were in the developmental stages of learning to read - Students at all levels of performance (i.e., At or Above Benchmark, Below Benchmark, and Well Below Benchmark) saw a dip in the 2020–2021 school year - The relative risks for being Well Below Benchmark were all greater than 1.0, indicating that for every grade and prior benchmark status, students were more likely to be Well Below Benchmark at the beginning of year in 2020–2021
[14]	To document children and adolescents’ perspectives regarding their social life and friendships during the COVID- 19 pandemic.	Social development assessed via semi-structured interview questions (e.g., What can you do with your friends even if you can’t see them in person? How do you feel since you can’t play/interact/ communicate in person with your friends?)	- Over half the participants said they were looking forward to going back to school because of the social aspects - Several participants noted the multiple opportunities for socialization that are created/facilitated by school attendance (e.g., opportunity to see friends daily, social opportunities outside of family) - A third no longer wish to use virtual means of communication post-pandemic and would prefer to only see their friends in person - More than half mentioned feeling that they are not really with friends during virtual contacts - Limitations and challenges with virtual communication (e.g., complexity of using virtual platforms for younger children, lack of spontaneity, loss of non-verbal communication for older children, lack of physical comfort) - Despite the significant limitations identified, most appreciated the fact that virtual communication allowed them to see or talk to friends
[32]	To assess the prevalence of child behavior, academic and sleep concerns, and parent stress and depression symptoms during COVID- 19, to test associations of parent-child wellbeing with child school format, and to examine effect moderation by child race/ethnicity and material hardship.	Social development assessed via peer problems subscale, and emotional development assessed via hyperactivity, conduct problems, emotional symptoms, and prosocial behaviours subscales measured via the Strengths and Difficulties Questionnaire (SDQ). Social development also explored via parents’ perceptions of how well children know their classmates and opportunities for friendships.	- Children receiving remote instruction had significantly higher hyperactivity and peer problems compared with children receiving in-person instruction (*p* < 0.005) and higher conduct problems compared with children receiving hybrid instruction (*p* < 0.10) - Compared with children receiving in-person instruction, parents of children receiving remote instruction were less likely to report that their child knew their classmates well (*p* < 0.0001), their child has enough opportunities to socialize (*p* < 0.0001), or that their child has a best friend (*p* < 0.10) - Parents of children receiving hybrid instruction were more likely to report that their child has enough socialization opportunities (*p* < 0.0001), or has a best friend (*p* < 0.0001) compared with children receiving in-person instruction - Non-Hispanic White children receiving remote instruction had significantly more hyperactivity (0.42–2.40) vs. children receiving in-person instruction - Children receiving hybrid instruction with material hardships had higher hyperactivity scores vs. children receiving remote instruction
[33]	To explore school-aged children’s and parents’ lived experiences of COVID- 19 and perceptions of its impact on psychological wellbeing in grade 4–6 students.	Social development assessed via interview questions (e.g., feelings during pandemic, lifestyle behaviours). Main theme that emerged was struggling with a lack of social interaction.	- Every child expressed sadness and frustration regarding the reduced amount and quality of social interaction with friends during COVID- 19, particularly during remote learning and lockdowns - Loneliness or missing friends was among the first things children mentioned when asked to describe their experience of COVID- 19 - Many children and parents recounted that a return to in-person learning, and subsequently, increased interaction with friends, led to elevated moods - Limited socializing prompted children to seek opportunities to interact with friends virtually, through video game chat functions and video calls, with several noting that children began using technology for the first time to socialize during COVID- 19 - Although several parents noted that virtual social interaction was not ideal and lacked the nuance and depth of face-to-face socializing, increased screen time was something many parents reluctantly allowed to facilitate children’s social needs
[34]	To evaluate the impacts of the COVID- 19 pandemic on parents and caregivers with young children.	Social and cognitive development assessed via interviews focussing on changes during the COVID- 19 pandemic. Themes centered around children’s (lack of) social development and schooling experiences.	- Lack of in-person socialization, isolation, and quarantine contributed to unmet social-emotional needs - Some children experienced heightened anxiety around new people and places due to lack of socialization, while other children experienced regressions in developmental milestones or their ability to manage developmentally appropriate responses - Many children fell behind during remote learning - Children who were English language learners or who experienced speech difficulties struggled to participate remotely - Parents with young children living with disabilities struggled to access resources and worried about regressions and impacts on speech development and social-emotional learning
[7]	To examine the impact of school disruptions on play-based learning during the pandemic and the lasting effect on classroom practices from the perspective of kindergarten educators.	Social development assessed via close-ended questions (e.g., limits to social interactions) and open-ended questions (e.g., social development). Cognitive development assessed via open-ended questions (e.g., gaps in academic skills).	- Students were more likely to play independently or near another student (i.e. parallel play), therefore, they had fewer opportunities to develop important socioemotional skills - Students lost opportunities to learn about basic social customs (e.g., sharing, cooperation, and compromise) and non-verbal social cues - Lack of consistent in-person learning led to minimal self-regulation, collaboration, problem-solving, and cooperative play skills - Masks created barrier to understanding others and to learning (e.g., difficulty modeling speech and sounds when teaching letters) - Students unable to access vital speech and language interventions or school readiness programs that they would have been able to access pre-pandemic
[35]	To investigate changes in children’s self-regulatory behavior before and during the COVID- 19 pandemic.	Emotional and cognitive development assessed via POCA which measures parents’ perceptions of child adaptation in the family context over the past 3 weeks, including the frequency of cognitive and behavioral inhibition, attention, task engagement, and hyperactivity.	- Significant within-child changes in self-regulation including poorer concentration, attention, task engagement and persistence, and greater impulsivity during the pandemic compared to pre-pandemic - Parenting stress during the pandemic was inversely related to children’s concentration (*r* = –0.40, *p* = 0.007), attention (*r* = –0.45, *p* 0.002), learning up to their ability (*r* = –0.34, *p* = 0.03), eagerness to learn (*r* = –0.49, *p* < 0.001), ability to work hard (*r* = –0.52, *p* < 0.001), and ability to wait their turn (*r* = –0.36, *p* = 0.01)
[12]	To capture the unique challenges and unanticipated successes associated with remote teaching and learning.	Social and emotional development assessed via interviews (e.g., socioemotional impacts of pandemic on students).	- Concerns around low socioeconomic students being unable to interact due to poor internet - Palpable loneliness felt from teachers through the screens, especially among children without siblings - Different emotional responses than normal (e.g., children who weren’t previously shy being shy, children who weren’t previously loud being loud) - Teachers worried about students who did not have the safe, social, or positive home environments that they were used to in the classrooms
[8]	To explore the changes to educators’ practices during the pandemic.	Social development assessed via interviews (e.g., school adaptations that impacted student outcomes).	- Educators described COVID‐19-related school changes as being primarily detrimental to children’s social development - Most social distancing changes were perceived as limiting social interactions and reducing the opportunities for social development - Changes caused less opportunity for students to practice crucial social skills (e.g., sharing materials, working in small groups for projects) and engaging in developmentally appropriate learning (e.g., cooperative groups) while also restricting their ability to engage with different cohorts/classrooms of students, impacting their social learning - Developmental impacts identified as having age specificity, with some adaptations more problematic than others depending on grade
[36]	This qualitative study explored teachers’ and parents’ perspectives on the impact of physical/social distancing and school closure policies on children’s socioemotional development.	Social and emotional development assessed via interviews focussing on the impact of COVID- 19 on children’s socioemotional and life-skills acquisition during and a comparison of children’s development before and after the COVID- 19 school closure.	- All parents highlighted social deprivation during the pandemic and socioemotional regression challenges in their children after the pandemic - All parents mentioned emotional issues among children (e.g., children being scared, apprehensive, sad, lost, stressed, increasingly cautious, less happy, and lack of emotional regulation abilities) during pandemic - Half of the parents discussed how their children went from being able to regulate their emotions and acting their age to being overly sensitive and reverting to behaviors that they previously grew out of - Because they were not socializing in-person for a prolonged time, teachers noticed troubles getting along in class (e.g., lack of sharing, ‘I want this now’ mentality)
[37]	To explore the perspectives of Canadian parents regarding the impact of COVID- 19 on social wellbeing during the first wave of the pandemic.	Social development assessed via interviews pertaining to participants’ experiences of COVID- 19 lockdowns and school closures for their family (e.g., social wellbeing and peer relationships).	- Children internalised physical distancing during the pandemic and how their strict adherence and anxiety about physical distancing presented barriers in interacting with peers - Perceived negative impacts of the pandemic ranged from children missing friends, to feeling lonely, to being deeply isolated - Parents highlighted concerns regarding social stagnation or regression - Reported barriers to receiving supports and services vital to social development during the pandemic including educational assistants, speech-language pathologists, and recreational

### Lack of socialization opportunities

Ten studies assessed students’ social development - including building relationships, sharing, cooperative play, listening, taking turns, proper social behaviour, and following directions - making this the most frequently assessed form of development [7,8,12,14,16,36,33,37,32,34]. Overwhelmingly, authors reported social development among elementary students suffered due to the pandemic limiting in-person socialization opportunities. Socially, this manifested as parent-reported changes to friendships (e.g., less ‘best’ friends reported, the need for virtual socialization), more positive attitudes towards in-person schooling among students (e.g., excitement about returning to in-person schooling as it offered an opportunity to socialize), and a teacher- and parent-reported decreases in socioemotional skill development among students (e.g., ability to share, cooperate, or participate in group work).

**Changes to Friendships.** When asked first-hand about their experiences of the pandemic, missing friends was among the first things elementary students mentioned [12,33]. Children’s friendships suffered through the pandemic as following the re-opening of some schools when parents had the option of in-person, hybrid, or fully remote learning, parents of children learning remotely (59.5%) reported their elementary students were less likely to know their classmates well, had insufficient opportunities to socialize with peers, and/or did not have a best friend when compared to those receiving in-person instruction [32]. Moreover, those receiving remote instruction reported higher peer problems than in-person counterparts and a decrease in the number of positive interactions with friends during the pandemic compared to prior [16,32].

With the limited in-person socialization opportunities, children sought out virtual interactions with friends, including videogame chat functions and video calling [33]. While most elementary students appreciated that virtual communication allowed them to see or talk to friends, many felt they were not really ‘with’ friends during virtual contacts and it was not an adequate replacement for in-person interactions as connecting virtually lacked the nuance and depth of face-to-face socialization [14,33]. Parents further noted limitations around virtual communication including the complexity of using virtual platforms for younger children, as it was many of their first times using technology for socialization. This meant that interactions largely lacked spontaneity, making the exchanges feel ‘forced’ and contributed to increased screen time for children [14].

**Shifting Attitudes Towards Schooling.** The lack of socialization opportunities and ability to see friends in-person meant that, for many elementary students, their attitudes towards attending school shifted and became more positive with a genuine excitement to return to in-person schooling. When participating in remote learning, most child participants were looking forward to going back to school because of the social aspects that are innate to attending school such as seeing friends daily and the ability to socialize beyond their immediate family [14]. Most children expressed a desire to stop using virtual socialization post-pandemic, instead expressing they would prefer to only see their friends in-person. In fact, both children and parents identified that the return to in-person learning, and subsequent increase in interactions with friends, led to elevated moods among elementary students.

**Decrease in Socioemotional Skill Development.** Together, isolation and a lack of in-person socialization with individuals outside of the immediate family left many children with unmet socioemotional needs, including learning how to behave in different social situations and understanding age-appropriate social norms (e.g., sharing, group work). Some children experienced heightened anxiety around new people and places, while others experienced regressions in developmental milestones or their ability to manage developmentally appropriate responses in social situations during the pandemic [34]. Teachers saw this manifest, attributing it to students largely missing their ability to practice basic social customs (e.g., cooperating, compromising) or opportunities to understand non-verbal social cues (e.g., someone being upset or feeling uncomfortable; [7,8]). This was exacerbated by the COVID- 19 policies in schools (e.g., social distancing), with teachers noting that children had less opportunity to use social skills (e.g., sharing materials, working in small groups for projects), students were required to use masks (e.g., missing the opportunity to observe non-verbal social cues), and students engaged less in developmentally appropriate learning (e.g., cooperative groups; [8]). Social learning was also inhibited as students were largely unable to engage with different cohorts/classrooms of students, which as been found to be particularly impacting on the social learning of elementary students [8]. Despite the removal of the pandemic public health policies, teachers reported some children showed lingering anxiety about proximity to others, presenting continued barriers to interacting with peers. For instance, many elementary students would opt to not participate in cooperative play, instead electing to play independently [7]. Teachers also reported that students had trouble getting along in class (e.g., ‘I want this now’ mentality). These pandemic-induced changes led to fewer opportunities to develop important socioemotional skills [7,36,37]. While many elementary students missed their friends during the pandemic and expressed a desire to get back to in-person school, upon returning, the social impacts of the pandemic were ever present and precluded many students from returning to a pre-pandemic ‘normal’ – thus presenting barriers to social development such as developing friendships, learning social norms, and developing age-appropriate social skills like sharing and working in groups.

### Emotional regression resulting from pandemic realities

Emotional development was assessed by five studies, highlighting emotional regulation, mental health management, self-awareness and regulation, conflict resolution, and emotion recognition [12,36,30,32,35]. Studies suggested that elementary students reverted to earlier stages of emotional development that, pre-pandemic, had been surpassed, including: (1) heighted impulsivity, sensitivity, and cautiousness compared to pre-pandemic [36,35]; (2) different emotional responses than what would have been ‘normal’ pre-pandemic (e.g., students who weren’t previously shy being shy, students who weren’t previously loud being loud; [12]; (3) a loss of age-appropriate emotional regulation abilities [36]; and (4) students reverting back to behaviors they had previously grown out of [36].

In understanding these emotional development challenges, it was suggested that some may have stemmed from pandemic policies, including mask wearing and remote instruction. One study found that mask wearing was related to lower emotion recognition accuracy in children, with older children who had more experience recognizing emotions pre-pandemic having higher emotion recognition accuracy than younger children who had less experience recognizing emotions pre-mask wearing [30]. Further, students who received remote instruction had significantly higher hyperactivity (compared to in-person instruction) and conduct problems (compared to hybrid instruction; [32]). Together, these results suggest students experienced some emotional regression during the pandemic.

### Cognitive gaps in learning

Four studies assessed students’ cognitive development, including factors related to attention, thinking, concentration, memory, and reading, writing, and communication abilities [7,31,34,35]. The consensus among these studies was that students fell behind in their learning during the pandemic, including both in their cognition-related behaviours and school achievement. Both parents and educators noted students were less able to concentrate, pay attention, be engaged with the task at hand, and be engaged with their learning when compared to before the pandemic [35]. Academically, during the pandemic, students performed poorer compared to pre-pandemic data [31,34]. Specifically, the impact of the pandemic on reading scores was found to be negative across grades K-6 and students at all levels of performance (i.e., At or Above Benchmark, Below Benchmark, and Well Below Benchmark) experienced a decline during the 2020–2021 school year compared to 2019–2020 [31]. These declines in reading scores are unsurprising, given that teachers highlighted the barriers faced in virtual learning environments coupled with mask wearing which makes modelling speech and sounds difficult when teaching literacy skills [7]. The disruptions to in-person elementary education impacted not only cognitive skills that support students’ learning, but also their academic achievement.

### Inequitable impacts on development

Research is clear that equity was an issue within the context of the pandemic [38]. Across disciplines, literature has underscored that the intersection of social determinants of health and wellbeing influenced how children, families, and communities survived, grew, and/or thrived throughout the pandemic [39]. Further it was highlighted within this review that these effects are moderated by stage of development, familial hardships, first languages, disability statuses, family socioeconomic statuses, safety in the home, and available social supports [12,30–32,34,35,37].

Many studies included in this review highlighted equity concerns among elementary students that were already largely established in both pre- and post- pandemic literature. For example, teachers noted a particular concern regarding the emotional regression of students who did not have safe or positive home environments [12]; children from families experiencing heightened social disruption had lower emotion recognition accuracy [30]; and students who were English language learners and from families experiencing socioeconomic challenges faced additional barriers to participating in remote schooling including language barriers and limited technology access (e.g., poor internet, malfunctioning devices; [34,12]). While these developmental impacts were further exacerbated by the pandemic and warrant recognition, these are long-standing equity issues within the elementary education system.

Equity concerns for elementary school students identified within this review centered around the critical importance of support services within the education system. Specifically, students faced barriers to receiving supports and services that were vital to their development during the pandemic including access to educational assistants, speech-language pathologists, and recreational therapists [37]. Further, students with disabilities struggled to access resources, vital early speech and language interventions, and school readiness programs that would have been available pre-pandemic- all of which would have helped to afford elementary school students a stronger academic start. The lack of access to basic services is of concern as parents and teachers alike have expressed those delays in accessing these services at age appropriate times resulted in not only speech and language regressions, but could negatively impact speech development for the child’s entire life [7,34]. Further, the negative impacts of the pandemic on reading scores were more pronounced in younger children (i.e., those in grades 1–3), who were in the key developmental phase of learning to read versus older children (i.e., those in grade 6; [31]). The lack of supports available to students during the pandemic beyond teachers and families meant that certain cohorts, namely students with disabilities and those who experienced the height of the pandemic during younger grades (i.e., grades 1–3), may be facing extended learning delays (e.g., literacy abilities; [7,34]). These various intersectional lenses through which children experienced the pandemic further exacerbated the already negative developmental effects displayed in children at large.

## Discussions

This scoping review provides the first overview of what we currently know regarding elementary students’ social, emotional, and cognitive development during the COVID- 19 pandemic in North America. Results from this review suggest that these three domains of development, social, emotional, and cognitive, were largely negatively impacted during the pandemic and that developmental effects were not experienced uniformly across all students. Socially, children suffered from the effects of isolation which led to changes within friendships, shifting attitudes towards schooling, and a decrease in key socioemotional skill development. Emotionally, many students regressed in their emotional regulation abilities, which may be attributed to mask wearing or remote instruction. Cognitively, students’ abilities and academic achievement were negatively impacted. Notably, children with disabilities and children in primary grades (1–3) faced heighted vulnerabilities due to the lack of supports available beyond teachers and parents, highlighting the importance of education-based support services that routinely go unrecognized, including educational assistants, speech and language pathologists, and recreational therapists.

Results from this review shed light on the impacts of social isolation among elementary students during the pandemic, particularly negative impacts on close friendships, shifting attitudes towards schooling, and declines in their socioemotional skill development. This is concerning, and yet not surprising, given the well-known negative effects of isolation on mental health. Brannen and colleagues [40] explored school-aged (i.e., 6–17 years) children’s mental health in the United States before and during the pandemic. After controlling for historical diagnoses and the rate of COVID- 19, the effect of pandemic-induced social isolation had a direct linear increase on the amount of anxiety among children, resulting in a 4-fold increase in pandemic social isolation-induced anxiety [40]. Further, mental health problems in childhood are associated with poorer mental health, physical health, life satisfaction, and quality of life in adulthood, meaning that the social isolation experienced during the pandemic could cause lifelong health impacts to elementary students if additional mental health supports are not provided post-pandemic [41]. Knowing that the rates of poor mental health increased among children in North America during the pandemic [42], child-centered socioemotional and mental health supports are needed, especially in schools where children spend most of their waking hours, to try and mitigate the long-term negative health consequences. In practice, this could include formal counselling sessions accessible to all students, with individual sessions focusing on student-specific needs while group sessions could foster socioemotional wellbeing among students. Longitudinal studies are also needed to track the development and mental health trajectories of children following the most severe bouts of school disruption experienced during the COVID- 19 pandemic to explore the long-term impacts of these adverse schooling experiences and to explore the potential benefits of child-centered socioemotional and mental health supports.

Pandemic-induced social isolation among students meant many of their attitudes towards in-person schooling positively shifted [14]. When students approach school with a positive attitude towards social interactions, the resulting connectedness is associated with positive outcomes including reduced emotional distress, suicidality, violence, and substance use [43–45]. These positive attitudes could serve as a protective factor for students that may serve to keep them in school. Specifically, Balfanz and colleagues [46] reported that school connectedness has positive influences on students’ academic success, wellbeing, and long-term learning outcomes, including a higher rate of school attendance, better academic performance, and better mental health. It is well-documented that students who are behind academically are more likely to drop out [47]; therefore, bolstering positive attitudes towards school and school connectedness among students may be one strategy to explore following the learning gaps caused by the pandemic to improve student retention, resilience, positive adaptation, and long-term academic outcomes. Health promotion programs that combine peer support and socioemotional learning, such as the LearnKind program – a free K-8, evidence-based program meant to foster socioemotional learning – may be one way to bolster the academic progression of students while fostering social connection among classmates.

Social and emotional regression among elementary students was noted as a key concern by parents and teachers alike. While these effects have been established and are more pronounced in younger cohorts of preschool children, wherein exposure to the COVID- 19 pandemic during the first year of life was associated with higher socioemotional development challenges [48], this review establishes that these effects continue to exist among older elementary students. Cooperative learning is a key skill that was highlighted as a gap in socioemotional development among students during the pandemic, which is particularly concerning given the importance of learning this skill for communication and interpersonal development, including the articulation of students’ thoughts, understanding others’ emotions, and the ability to positively interact with other students [49]. Together, the magnitude of this issue is concerning as the issue is present among preschool and elementary school students during the pandemic. Given that students who were preschoolers during the pandemic have now begun to enter the formal education system with these socioemotional developmental delays, these delays are likely to continue permeating elementary education. The reality is that this problem will persist, highlighting that classrooms are in dire need of ongoing financial and personnel support following the pandemic to bolster children’s socioemotional development as positive development in these domains provides a critical foundation for lifelong development and learning (US Department of Health and Human [50]). As evidenced by the results of this review, without age-appropriate socioemotional development, children may be lacking the tools to meet the cognitive milestones of elementary curriculums.

Meeting the cognitive milestones of elementary school was particularly challenging during the pandemic, with delays in both cognitive skills and academic achievement being highlighted by this review. Specific cognitive skill deficits were emphasized by teachers and parents, including attention, thinking, concentration, memory, and reading, writing, and communication abilities [7,31,34,35]. Research underscores how variation in cognitive measures, such as those listed above, predicts performance on various tasks that are important throughout the life course including comprehension, following directions, solving problems, and taking notes [51,52]. Together, these cognitive abilities are directly associated with academic performance [53]. Importantly, studies suggest that given this direct association, focussing on the development of cognitive abilities, rather than direct achievement, may be a better avenue when supporting students to meet their learning objectives [53]. What remains unclear from the current body of research is what the cognitive decline noted in students was due to. Future longitudinal studies should explore the possible mechanisms behind this decline, including reasons such as lack of teacher interaction versus screen fatigue during periods of emergency remote learning. Following the pandemic wherein both cognitive skill development and academic achievement were impacted, focussing on bolstering the cognitive abilities of students may best position students to academically succeed. This may mean that elementary classrooms require support to adapt to the differing needs of students within the same grade-level classroom working at different abilities.

Despite the COVID- 19 pandemic impacting all children, these impacts were not experienced uniformly. Namely, intersecting aspects of children’s lives, including the age at which they experienced the pandemic and pre-existing disabilities, heightened the negative developmental effects of the pandemic, further marginalizing these children. Calls to action to the government, the education system, community organizations, and health professionals surrounding the disproportionate effects of the pandemic on children’s development among previously marginalized groups (i.e., groups who are intentionally or unintentionally excluded from access to power and resources, and who are perceived as marginal, less important, or less valued within dominant societal structures; [54]) were made by researchers and community organizations at the outset of the pandemic [55]. Despite these calls to action, results from this review suggest that the developmental impacts of the pandemic were heightened among these marginalized groups who already faced greater health disparities than non-marginalized peers. Post-pandemic efforts, such as policy reforms, should be centered around marginalized children, specifically those who are at risk of prolonged developmental delays as highlighted by this review, to support their positive development while accounting for the intersecting aspects of their lives that may place them at a disadvantage compared to their non-marginalized peers.

## Limitations

This review is not without limitations. Most of the included studies present cross-sectional data, thus limiting our ability to infer causation and reducing the generalizability of findings as they only captured children’s experiences at one point during the pandemic. Given the developmental effects of the pandemic noted in the included articles, this calls for longitudinal, post-pandemic studies to explore if these effects are enduring, and what supports students, families, and teachers need following pandemic disruptions. Further, nearly half (*n* = 6) of the included studies did not provide data on the gender of children, limiting the generalizability of findings. Since male and female children experience different development milestones, future studies should explore the gendered and intersectional effects of the pandemic on development, if any, to ensure that programs and policies are developed to meet the needs of children based on their intersecting identities (e.g., age, gender, ethnicity, family socioeconomic status). In addition, the studies included in this review largely discussed development from a deficit perspective, versus a strengths-based perspective. While this could indicate the deleterious developmental impacts of the pandemic, focussing on the potential resilience factors fostered during the pandemic, such as those stemming from increased family engagement or the development of technological skills, could provide a more balanced understanding of pandemic-related changes in elementary children. Finally, many of the studies consisted of parents or educators reporting on behalf of elementary school children, thus introducing the possibility of proxy reporting bias. Future research should strive to including children *in* research versus conducting research *on* children to ensure we are accurately capturing children’s experiences and developmental trajectories.

## Conclusions

Elementary school students struggled developmentally during the pandemic, experiencing social, emotional, and cognitive impacts. Social implications included difficulties building relationships, sharing, participating in cooperative play, listening, taking turns, proper social behaviour, and following directions. Emotional implications included challenges with emotional regulation, mental health management, self-awareness and regulation, conflict resolution, and emotion recognition. Lastly, cognitive implications included deficits in attention, thinking, concentration, memory, and reading, writing, and communication abilities. Children highlighted the impacts to their friendships as the biggest concern, whereas parents and teachers reported the learning gaps among students as the largest concern. Namely, this review highlighted how children with pre-existing disabilities were lost in the rapid shuffle from in-person to emergency remote instruction. While focussing on the transition at the elementary school system level, these children with learning difficulties were largely left behind. School-based supports to bolster the development of all children, but particularly those experiencing multiple intersecting disparities, must be developed and implemented to better support this population post-pandemic. By supporting the positive development of elementary children, their lifelong health and wellbeing may be improved.

## Supporting information

S1_Checklist Preferred Reporting Items for Systematic reviews and Meta-Analyses extension for Scoping Reviews (PRISMA-ScR) Checklist. (DOCX)

S1_Table Database Search Strategy. (DOCX)
