# Supplementary material for: Elementary students’ social, emotional, and cognitive development during the COVID- 19 pandemic in North America: A scoping review
Source: PLOS Glob Public Health. 2025 Sep 11;5(9):e0005148. doi: 10.1371/journal.pgph.0005148 (PMC12425295; doi:10.1371/journal.pgph.0005148)
Supplement: S1_Table — (DOCX) [file pgph.0005148.s002.docx]

**S1 Table.** *Database Search Strategy*

| **Concept** | **Search** |
| --- | --- |
| Elementary students | "elementary student*".mp. or Students/ or Schools/ or "elementary school*".mp. or "grade school".mp. |
| Development | exp Child Development/ or "child development".mp. or “social development”.mp. or “emotional development”.mp. or “cognitive development”.mp. |
| COVID-19 Pandemic | "COVID-19 pandemic".mp. or exp COVID-19/ |
